# The Impact of a Health Coaching App on the Subjective Well-Being of Individuals With Multimorbidity: Mixed Methods Study

**DOI:** 10.2196/78738

**Published:** 2026-02-04

**Authors:** Isabelle Symes, Alexandra Burton, Daniela Mercado, Feifei Bu

**Affiliations:** 1 Department of Behavioural Science and Health, Institute of Epidemiology and Health Care, University College London, 1-19 Torrington Place, London, WC1E 7HB, United Kingdom, 44 (0)20 7679 1720; 2 Centre for Preventive Neurology, Wolfson Institute of Population Health, Queen Mary University of London, London, United Kingdom; 3 Centre for Psychiatry and Mental Health, Wolfson Institute of Population Health, Queen Mary University of London, London, United Kingdom; 4 Holly Health, London, United Kingdom

**Keywords:** multimorbidity, health coaching app, digital health intervention, subjective well-being, mixed methods, mechanisms of action

## Abstract

**Background:**

Multimorbidity, the coexistence of 2 or more chronic conditions, is associated with poor well-being. Health coaching apps offer cost-effective and accessible support. However, there is a lack of evidence of the impact of health coaching apps on individuals with multimorbidity.

**Objective:**

This study aimed to assess the impact and acceptability of a health coaching app (the Holly Health [HH] app) on the subjective well-being (SWB) of adults with multimorbidity.

**Methods:**

This study used an explanatory-sequential mixed methods design, with quantitative secondary data analysis in the first phase and qualitative interviews in the second phase. In the quantitative phase (n=565), pre- and post-SWB (Office for National Statistics' 4 personal well-being questions [ONS4]) scores from existing app users with multimorbidity were analyzed using Bayesian growth curve modeling to assess the impact of HH. In the qualitative phase (n=22), data were collected via semistructured interviews and analyzed using reflexive thematic analysis. Mechanisms of action that supported SWB were categorized using the Multi-Level Leisure Mechanisms Framework.

**Results:**

There was a significant increase in life satisfaction (Coef.=0.71, 95% highest density interval [HDI] 0.52‐0.89), worthwhileness (Coef.=0.62, 95% HDI 0.43‐0.81), and happiness (Coef.=0.74, 95% HDI 0.54‐0.92) and a decrease in anxiety (Coef.=−0.50, 95% HDI −0.74 to −0.25) before and after using the HH app. Overall, 8 acceptable app features activated 5 mechanisms of action, including behavioral, psychological, and social mechanisms. Three additional factors influenced the acceptability of the health coaching app: type of chronic condition, availability of time, and the use of other support tools.

**Conclusions:**

The study demonstrates that health coaching apps could be effective and acceptable support tools for individuals with multimorbidity. This study contributes to understanding why health coaching apps support SWB and could be used to inform the development of future digital health interventions in multimorbidity.

## Introduction

By 2035, it is projected that two-thirds of adults in England aged 65 years and older will have multiple chronic conditions, with a 50% increase in people developing 4 or more chronic conditions [1]. Multimorbidity is the coexistence of 2 or more long-term physical or mental health conditions [2]. It has been associated with key disease clusters including hypertension, diabetes, asthma, cancer, depression, and anxiety [3]. Multimorbidity is more prevalent among older adults, females, and those living in lower-income areas [4,5]. It is also influenced by socioeconomic (eg, education), behavioral (eg, smoking), psychosocial (eg, loneliness), and biological factors [6].

Due to the complex nature of multimorbidity, consequences range in severity and scope. Associated outcomes include premature mortality [7], disability [8], elevated frailty risk [9], and hospitalization [10]. Multimorbidity also contributes to economic burden, including increased health care use and costs [11]. The complex health care needs associated with multimorbidity management cannot be met sufficiently by current health care systems with a single disease focus [12]. There is a general lack of holistic support options, especially an inattention to well-being in primary care consultations [13]. Emphasis on patient-centered approaches, where individuals can establish and work toward their own health and well-being goals, is essential to the effective management of multimorbidity [14]. Patient-centered care is a key component of the National Institute for Health and Care Excellence (NICE) guidance on multimorbidity [15].

Subjective well-being (SWB) is defined as an individual’s thinking and feeling toward life as desirable [16]. It encompasses 3 dimensions: evaluative, eudaemonic, and affective [17,18]. Evaluative well-being refers to the overall assessment of satisfaction with life, eudaemonic well-being links to accounts of the meaning of life, and affective well-being reflects feelings and moods experienced every day [19]. Previous research has consistently shown the association between multimorbidity and poor well-being [6]. This is concerning, given SWB could play an important role in health maintenance and management. For example, increased SWB has been found to predict greater engagement in health-promoting behaviors [20], which has significant implications for multimorbidity management [21].

In the last few decades, there has been an increasing recognition of engagement in leisure activities, such as physical activity, as a protective factor against multimorbidity [22]. The Multi-Level Leisure Mechanisms Framework [23] suggests that engaging in leisure activities elicits various health benefits via psychological (eg, improved well-being), biological (eg, increased physical fitness), social (eg, increased social support), and behavioral (eg, increased motivation) mechanisms. This is supported by empirical research showing that regular exercise (behavioral mechanism) and strong social networks (social mechanism) are associated with a lower risk of multimorbidity [24], while increased social support (social mechanism) and lifestyle changes (behavioral mechanism) are associated with slower illness progression [25] and improved life expectancy [26].

Digital solutions can be used to promote these positive behavioral changes, such as increased engagement in physical activities, which can then subsequently enhance subjective and physical well-being, with health coaching apps offering cost-effective and accessible support options [27]. Health coaching apps are a form of digital health intervention (DHI), which are tools that use information and communication technologies for the improvement of health management, monitoring, prevention, treatment, and lifestyle [28]. Various studies have demonstrated the feasibility, acceptability, and effectiveness of the use of health coaching apps to support the self-management of common chronic conditions such as depression [29], diabetes [30], and hypertension [31]. However, there is a paucity of evidence exploring the mechanisms of their impacts. Moreover, evidence of their impacts on individuals with multimorbidity remains preliminary and limited, particularly with interventions that explicitly target SWB as a primary outcome [32]. To our knowledge, there are currently no studies specifically focusing on the impact of digital interventions on the SWB of people with multimorbidity. Therefore, this study focused on well-being, given its established association with multimorbidity and as it remains underexplored compared to more commonly studied outcomes (eg, quality of life).

This study aimed to assess the impact of a health coaching app on the SWB of people with multimorbidity. More specifically, it addressed the following research questions: (1) Does using a health coaching app have a positive impact on the SWB of people with multimorbidity? (2) If a positive impact is observed, which app features contribute to improved SWB for people experiencing multimorbidity and how do they support SWB? (3) Which factors influence the perceived acceptability of the app?

## Methods

### Holly Health App

This study is partnered with Holly Health (HH), a health coaching app supported by iOS, Android, and web [33]. Key features include daily chatbot support, nudges and reminders, mood, stress, and energy tracking, habit tracking, health reviews, tailored educational content, and short-term challenges (see Multimedia Appendix 1 for app images). These challenges typically involve 1-week goals (eg, mindfulness or physical activity challenges) that are ranked from easy to hard. The app targets well-being, sleep, exercise, and eating to promote healthy aging and support chronic condition management. Engagement with the app is user-directed, allowing individuals to interact with different features according to their needs and preferences rather than through a prescribed pathway. HH is supported by the UK National Health Service (NHS) Innovation Accelerator, which aims to support high-impact innovations that address NHS priorities [34]. Currently, the app is partnered with more than 200 general practitioner (GP) practices in the United Kingdom. Users typically access the app through GP practices, where they are invited via an SMS text message to download and use the app free of charge. HH is grounded in cognitive behavioral therapy (CBT) [35], acceptance and commitment therapy [36], mindfulness, and the small habits approach, based on frameworks such as the Capability-Opportunity-Motivation Behavior (COM-B) model [37]. While HH incorporates features to support chronic condition management, it was not specifically developed for people with multimorbidity.

### Study Design

**Figure 1.**
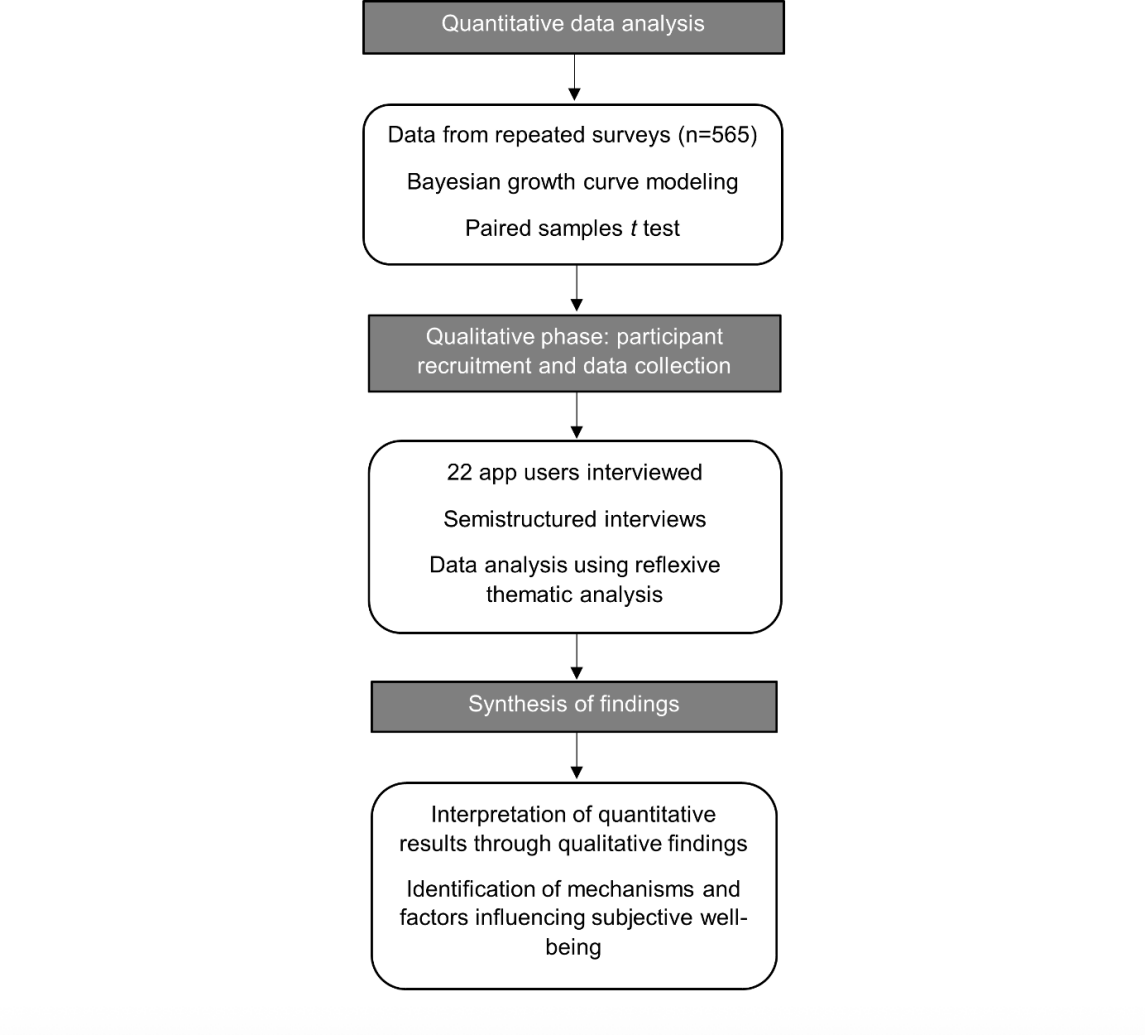
Study design flowchart.

This study used an explanatory sequential mixed methods design [38], comprising a quantitative phase followed by the qualitative phase (see Figure 1). First, the quantitative phase involved secondary data analysis of repeated survey data (n=565). This was followed by the qualitative phase, where semistructured interviews were conducted with 22 app users. The two phases were conducted and reported sequentially, with integration occurring during the interpretation of findings to explain and expand on the quantitative results. This study defines multimorbidity as 2 or more chronic conditions. Guidance for secondary data analysis (STROSA: Standardized Reporting of Secondary Data Analyses [39]) and qualitative research (COREQ: Consolidated Criteria for Reporting Qualitative Research [40]) were followed.

### Ethical Considerations

The University College London (UCL) Research Ethics Committee approved this study (26737/001). All participants provided informed consent prior to participation, and all procedures were conducted in accordance with the ethical standards and institutional regulations. Secondary quantitative data were fully anonymized by HH before being transferred to the researchers for analysis. Data were stored on a secure research environment provided by UCL, accessible only to the research team. The interview recordings and transcripts from the qualitative phase were transferred to a secure research environment, ready for checking and deidentification. HH provided £5 (US $6.75) Amazon vouchers to those who completed an interview.

### Participants

#### Quantitative Phase

Secondary data were obtained from health surveys of app users conducted by HH between March 2023 and March 2024. The surveys were originally collected outside the app but were later integrated within the app platform. Eligibility criteria for this study required participants to (1) report 2 or more chronic conditions during the onboarding health survey, (2) be aged 18 years or more, and (3) complete the baseline and follow-up (≥8 weeks) SWB questionnaires (n=565). Completion of the baseline questionnaire was compulsory; however, demographic information questions (eg, age, gender, and ethnicity) and the follow-up questionnaire were optional. Before applying the eligibility criteria, 882 app users had completed the baseline and follow-up questionnaire. Of this, 565 app users were eligible (64.1% of the available data). The sample size meets the minimum sample size to yield an alpha of .05, a statistical power of 0.8, and a small effect size (*d*=0.2) [41] in SWB outcomes. The assumption of a small effect size was based on previous literature that investigated the influence of a DHI on the well-being of individuals with a single chronic condition [42]. Demographics such as age, gender, and ethnicity were captured through the app, which were only available for 55.2% (312/565) of participants. Missingness occurred because the SWB survey was not initially integrated into the app; therefore, it cannot be linked to demographic information.

#### Qualitative Phase

A criterion-based purposive sampling technique was used to identify eligible app users with multimorbidity who had used the app for at least 2 weeks. Recruitment was conducted via an email campaign distributed by the HH team to eligible app users, and participants self-selected to take part by scheduling an interview through a Calendly link. The first email was sent to 2190 app users. A total of 30 app users replied, and interviews were booked; however, 9 did not go ahead due to participants’ scheduling conflicts and technical difficulties. The email was then sent to 40 new app users who met the criteria, resulting in 1 more interview and 22 interviews in total. Information power was referenced during the planning and data collection phases to evaluate the sufficiency of the sample size [43]. This process was iterative, with no predefined number of interviews. Given the broad aim of the study and the use of cross-case analysis alongside the application of an established theory and a specific sample, a moderate sample size was deemed appropriate.

### Materials and Procedure

#### Quantitative Phase

The primary outcome was measured by the Office for National Statistics’ 4 personal well-being questions (ONS4) [44]. The 4 questions cover life satisfaction (evaluative), worthwhileness (eudemonic), happiness (affective), and anxiety (affective), each measured on an 11-point Likert scale (0‐10) (see Multimedia Appendix 2 for questions and scoring). The ONS4 data were collected at baseline and the 8-week follow-up with no time limit for completion. The median number of days between baseline and follow-up completion was 84.5 (IQR 59-160) (~12 weeks). Other relevant data included age (18‐34, 35‐54, 55‐64, and 65+ years), gender (male or female), ethnicity (White and other), number of daily habits completed (eg, 10 minutes of mindfulness=1 habit), number of medical conditions, and whether the app user felt they had developed automatic habits without relying on the app (yes or no). Chronic conditions were recorded through self-report using a predefined checklist. If a condition was not represented, app users had the option to select “something else.” The checklist was developed based on publicly available NHS data on condition prevalence and the most commonly diagnosed chronic conditions identified among the first 20,000 HH users. They were not able to provide any additional free-text responses.

#### Qualitative Phase

Potential participants were sent an information sheet and a Calendly link to an interview booking page by HH via email. Participants could book 45-minute time slots for the interview. To book the time slot, participants were required to read the information sheet and check all the consent boxes, providing consent to take part in the study. The interviews were recorded and transcribed via Microsoft Teams. Due to confidentiality, we cannot confirm whether the interview participants were also part of the survey dataset. All interviews were conducted by the lead researcher (IS). The interviews were semistructured, following a topic guide (see Multimedia Appendix 3) discussing participants’ multimorbidity and positive and negative experiences of using HH, with the flexibility of follow-up questions and prompts. The topic guide was developed by the lead researcher (IS), with supervision from 2 coauthors (FB and AB). It was informed by the study aims and the conceptual underpinnings of HH. The topic guide was piloted, with one question subsequently divided into two to improve clarity. This interview was included in the analysis. Demographic information, including self-reported chronic conditions, was collected during the interviews. The mean duration of interviews was 24.68 minutes (range 12.31-52.34 minutes). A distress protocol was in place due to potentially sensitive topics being disclosed, informed by the Qualitative Research Distress Protocol tool [45].

A reflective log was used by the lead researcher (IS) to guide self-reflection and transparency during the research process [46]. IS is a White female researcher (MSc) with experience in conducting research with people with long-term conditions and semistructured interviewing. Independence from HH was clarified to participants before the interview to ensure transparency and reduce potential bias. IS’s values of respect, transparency, and evidence-based practice fostered rapport and shaped expectations of the app. IS’s positionality, shared with many participants who were predominantly White and female, required reflexive attention to how shared identity could influence assumptions. Reflexive journaling and supervision supported critical reflection on how personal characteristics, interviewing style, and emotional responses influenced both data collection and analysis.

### Analyses

#### Quantitative Phase

Descriptive analyses were conducted to summarize characteristics, including frequencies, percentages, means, and SDs. To examine changes in SWB before and after the intervention, quantitative data were analyzed using Bayesian growth curve modeling. It allowed us to examine person-specific and average changes between 2 time points, with random intercept and slope. For the main analysis, we fitted an unconditional linear growth model to the full sample (n=565) separately for each of the ONS4 SWB measures, using noninformative priors, 2000 iterations, 6 chains, a burn-in of 1000, and a thinning of 5. Please see Multimedia Appendix 4 for technical details. A paired samples *t* test was conducted as sensitivity analysis, using both the full sample and a conditional sample restricted to complete demographic data (n=312) to assess the potential influence of missing data. Sensitivity analyses were also conducted to examine if changes in SWB differed by individual characteristics including age, gender, ethnicity, number of medical conditions, automatic habits, and number of habit completions. This was done by fitting separate conditional growth models for each demographic covariate using data from a reduced sample with valid data for that specific covariate (n=292 to 312). The Bayesian growth curve models were fitted in R 4.3.2 (R Foundation for Statistical Computing) and using Markov Chain Monte Carlo algorithms, implemented in JAGS. Model convergence was assessed using the Gelman-Rubin statistic and visual inspection of the posterior distributions. Paired sample *t* tests were conducted in IBM SPSS Statistics 28.0 for Microsoft Windows.

#### Qualitative Phase

Transcripts were analyzed using NVivo 12 (Lumivero). Reflexive thematic analysis was used, a theoretically flexible methodology used to understand experiences and behaviors [47,48]. The analysis adopted an essentialist, experiential, inductive approach to capture and reflect participants’ direct experiences and perspectives [49]. Once the themes were fully developed inductively, they were mapped onto the Multi-Level Leisure Mechanisms Framework [23] top-level categories and were interpreted in the context of participants’ reported experiences with HH. This framework was selected as it offers a clear structure for understanding (eg, psychological, social, and behavioral mechanisms of change), which aligns with the study’s aim of identifying which app features may support SWB and the factors linked to acceptability. The framework did not shape the coding or theme development process; rather, it was used post hoc to organize the themes within established mechanisms. This supported subsequent interpretation of findings, enabling connections to be drawn between themes and app features.

The lead researcher (IS) coded the transcripts twice, followed by discussion with two coauthors (FB and AB), who each read 2 transcripts. The reflective log was referred to throughout the analysis, with potential preconceptions about the familiarity of using technology considered.

### Synthesis of Findings

This study followed an explanatory sequential mixed methods design, in which synthesis of findings occurs during the interpretive stage. In the quantitative phase, the analysis identified both the direction and significance of change in SWB. The qualitative phase was undertaken to provide explanatory depth and contextualization, focusing on potential mechanisms and specific app features that may be associated with the observed findings. The interpretation of the quantitative findings through consideration of the qualitative evidence is presented in the Discussion section.

## Results

### Quantitative Results

#### Sample Characteristics

Among app users with valid demographic information (n=312), 35.9% (n=112) were aged 65+ years, forming the largest age category (see Table 1). Over three-quarters (n=245, 78.5%) were female, and 94% (n=280) were White.

**Table 1.** Quantitative characteristics.

Variables	Values
Sex (n=312), n (%)	
Female	245 (78.5)
Male	67 (21.5)
Age in years (n=312), n (%)	
18‐34	14 (4.5)
35‐54	84 (26.9)
55‐64	102 (32.7)
65+	112 (35.9)
Ethnicity (n=298), n (%)	
White	280 (94)
Other (Asian, Black, Mixed, or Other)	18 (6)
Automatic habits^a^ (n=292), n (%)	
Yes	219 (75)
No	73 (25)
Number of medical conditions (n=312), mean (SD)	4.64 (3.36)
Habit completions^b^ (n=312), mean (SD)	161.86 (229.51)

a Have you started to do any of your habits automatically? That is, without relying on the app to remind you?

b Number of habits recorded as completed.

The mean number of chronic conditions recorded was 4.64 (SD 3.36; Table 1). As shown in Table 2, the most common conditions were anxiety (126/312, 40.4%), hypertension (122/312, 39.1%), depression (108/312, 34.6%), and arthritis (96/312, 30.8%).

**Table 2.** Chronic condition frequency (n=312).

Chronic condition	Values, n (%)^a^
Mental health	
Anxiety	126 (40.4)
Depression	108 (34.6)
Cardiometabolic	
Hypertension	122 (39.1)
High cholesterol	85 (27.2)
Type 2 diabetes	43 (13.8)
Prediabetes	38 (12.2)
Heart disease	25 (8.0)
Musculoskeletal or pain	
Arthritis	96 (30.8)
Joint mobility issues	83 (26.6)
Chronic pain	41 (13.1)
Fibromyalgia	16 (7.1)
Osteoporosis	20 (6.4)
Severe mobility impairments	13 (4.2)
Respiratory	
Asthma	58 (18.6)
Chronic obstructive pulmonary disease	18 (5.8)
Gastrointestinal	
Irritable bowel syndrome	28 (17.2)
Irritable bowel syndrome or inflammatory bowel disease	19 (7.2)
Other	
Something else	104 (33.3)
Insomnia	52 (19.6)
Fatty liver	14 (9.2)
Stroke	15 (4.8)
Long COVID	12 (3.8)
Kidney disease	9 (2.9)

a Valid percentages calculated based on the number of app users who responded to each condition item. App users could report multiple chronic conditions.

#### Bayesian Growth Curve Model

**Figure 2.**
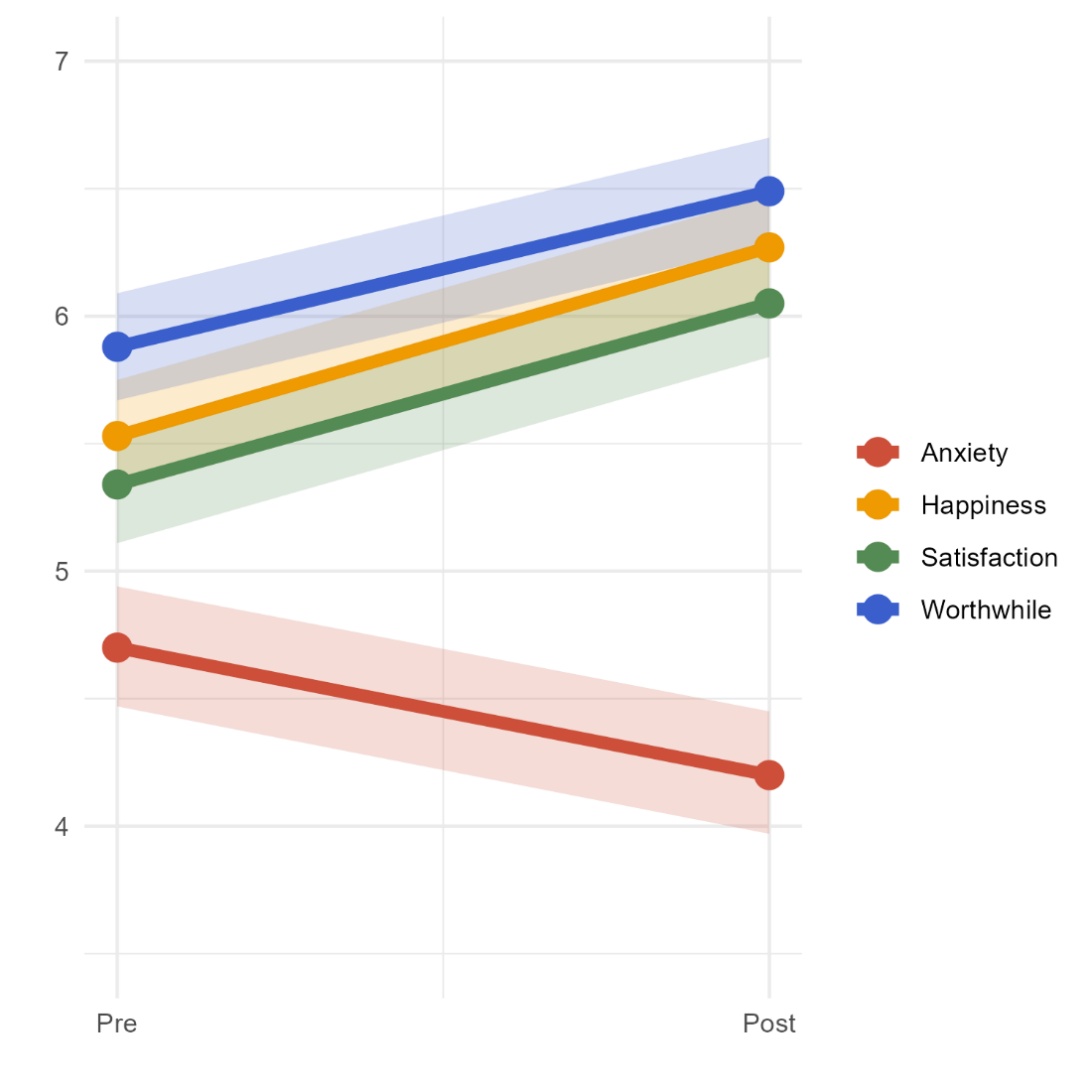
Bayesian growth curve model mean trajectories.

The results from the unconditional Bayesian growth curve models are shown in Figure 2. On average, life satisfaction increased by 0.71 (95% highest density interval [HDI] 0.52-0.89) after using the app for 8 or more weeks, worthwhileness increased by 0.62 (95% HDI 0.43-0.81), and happiness increased by 0.74 (95% HDI 0.54-0.92). Alongside this, anxiety decreased by 0.50 (95% HDI −0.74 to −0.25).

#### Sensitivity Analyses

As shown in Table 3, paired samples *t* tests revealed a significant difference in life satisfaction (*t*_564_=−7.65, *P*<.001), worthwhileness (*t*_564_=−6.58, *P*<.001), happiness (*t*_564_=−7.46, *P*<.001), and anxiety scores (*t*_564_=3.87, *P*<.001). This finding aligns with the Bayesian growth curve model, again suggesting a significant improvement in all ONS4 domains.

**Table 3.** Paired samples *t* test results (n=565).

ONS4^a^ domains	Mean (SD)	*t* test^b^	*P* value	Cohen *d*^c^
Life satisfaction^d^		–7.65	<.001	–0.32
Prescore	5.34 (2.60)			
Postscore	6.06 (2.42)			
Worthwhileness^d^		–6.58	<.001	–0.28
Prescore	5.88 (2.55)			
Postscore	6.50 (2.46)			
Happiness^d^		–7.46	<.001	–0.31
Prescore	5.54 (2.64)			
Postscore	6.28 (2.53)			
Anxiety^e^		3.87	<.001	0.16
Prescore	4.70 (2.89)			
Postscore	4.20 (2.93)			

a ONS4: Office for National Statistics’ 4 personal well-being questions.

b Two-tailed *t* test (*df*=564).

c Cohen *d *(1988) effect sizes: small (*d*=0.2), medium (*d*=0.5), and large (*d*=0.8).

d Life satisfaction, worthwhileness, and happiness thresholds: low (0-4), medium (5-6), high (7-8), and very high (9-10).

e Anxiety threshold: very low (0-1), low (2-3), medium (4-5), and high (6-10).

In the conditional Bayesian growth curve models (n=292 to 312), group effects were tested (see Multimedia Appendix 5). We found some evidence that age, automatic habits, and number of habit completions were associated with SWB outcomes at baseline. However, there was little evidence that these variables were related to the rate of change for any of the SWB measures.

### Qualitative Results

#### Sample Characteristics

Participants in the qualitative sample (n=22) were aged 29‐73 years (mean age 55.6 years). Most were female (n=18, 81.8%) and White (n=19, 86.4%). The most common chronic conditions reported were high blood pressure (n=7), anxiety (n=6), and type 2 diabetes (n=6) (see Table 4). Participants described varying levels of engagement with HH, from ongoing active use to reduced or discontinued use.

**Table 4.** Qualitative sample characteristics (n=22).

Characteristics	Values
Sex, n (%)	
Female	18 (81.8)
Male	4 (18.2)
Age (years), n (%)	
18‐34	1 (4.6)
35‐54	6 (27.3)
55‐64	10 (45.5)
65+	5 (22.7)
Ethnicity, n (%)	
White	19 (86.4)
Other (Asian, Black, or prefer not to say)	3 (13.6)
Chronic conditions, n	
High blood pressure	7
Anxiety	6
Type 2 diabetes	6
Depression	4
High cholesterol	4
Fibromyalgia	3
Asthma	2
Chronic fatigue syndrome	3
Diverticulitis	2
Irritable bowel syndrome	2
Osteoarthritis	2
Prediabetes	2
Sciatica	2
Stroke	2
Other^a^	39

a Atrial fibrillation, attention-deficit/hyperactivity disorder, borderline osteoporosis, bowel cancer, bowel problems, chronic pain, cochlear hydrops, complex posttraumatic stress disorder, congenital heart disease, coronary heart disease, diabetic neuropathy, Graves’ disease, gout, hard of hearing, heart disease, heart valve problems, hepatitis, hypermobility spectrum disorder, hypermobility syndrome, hypertension, inflammatory bowel disease, long COVID, low iron, mobility problems, multiple myeloma, musculoskeletal problems, perimenopausal disorder, peripheral neuropathy, rheumatoid arthritis, sleep apnea, tinnitus, thrombocytopenia, and unstable bladder.

#### Mechanisms of Action

##### Summary

**Figure 3.**
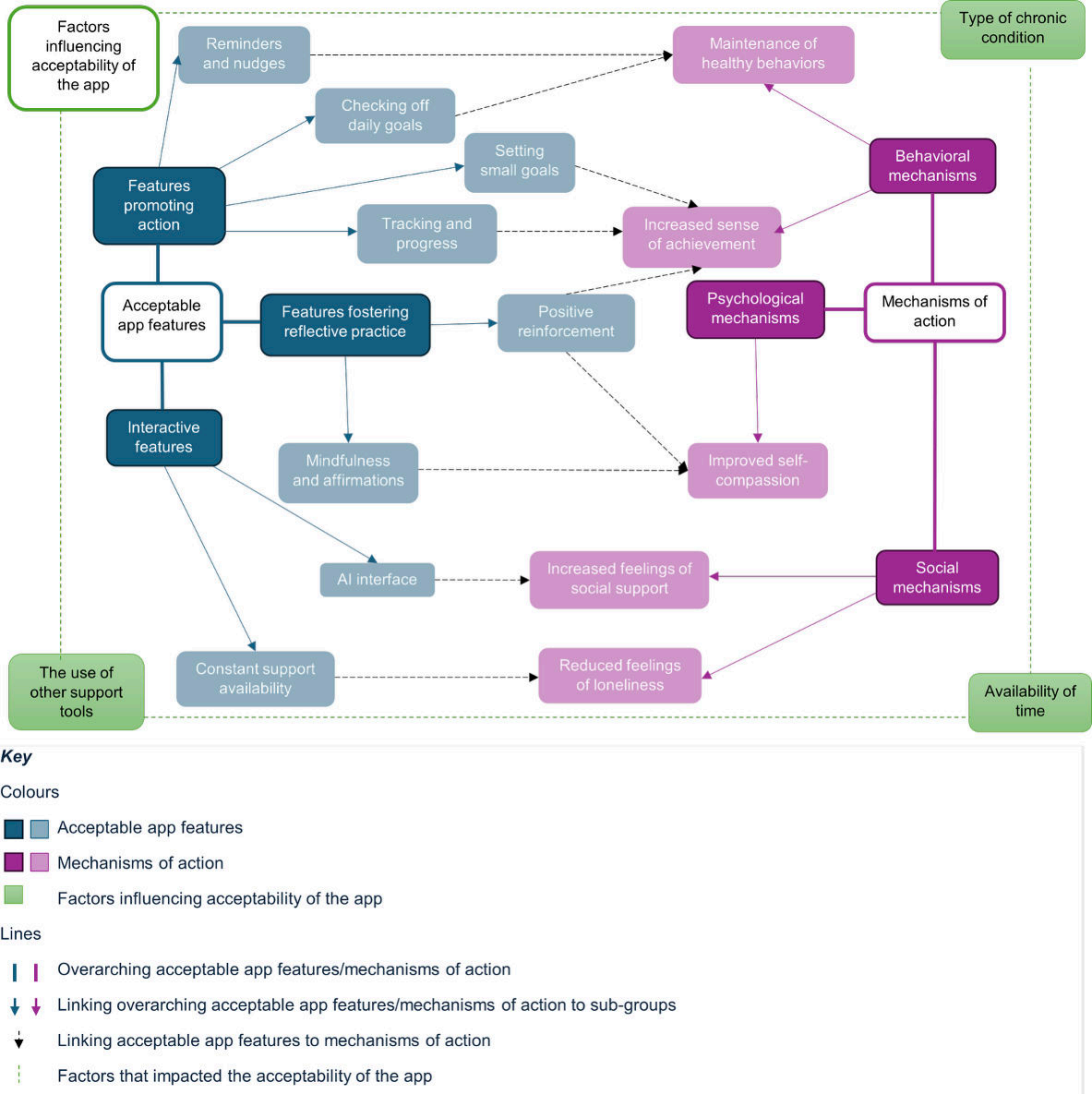
Thematic associations diagram. This figure illustrates the relationship between app features and the mechanisms of action identified in the study. The figure reflects both overarching categories from the Multi-Level Leisure Mechanisms Framework and participants' reported experiences. Dark pink denotes overarching mechanisms, while light pink indicates specific submechanisms. Similarly, dark blue denotes specific features within these groups. The black dotted arrows indicate the key associations between app features and mechanisms. Factors influencing app acceptability are shown in green, surrounding both features and mechanisms. AI: artificial intelligence.

Participants described 8 app features that activated 5 mechanisms of action leading to perceived improvements in SWB. App features included (1) those promoting action, such as “checking off daily goals,” “setting small goals,” “reminders and nudges,” and “tracking and progress reports”; (2) features fostering reflective practice, including “mindfulness and affirmations” and “positive reinforcement”; and (3) interactive features such as “AI interface” (AI: artificial intelligence) and “constant support availability.” Behavioral mechanisms included “maintenance of healthy behaviors” and “increased sense of achievement.” The psychological mechanism was “improved self-compassion,” and the social mechanisms included “increased feelings of social support” and “reduced feelings of loneliness.” The acceptability of the app was impacted by “type of chronic condition,” “availability of time,” and “the use of other support tools.” These relationships are presented in Figure 3. Participant quotes are used to illustrate the relationship between the app features and the activated mechanisms of action.

##### Behavioral Mechanisms

###### Maintenance of Healthy Behaviors

To maximize the benefits of engaging in healthy habits, including improved SWB, participants needed to reach a point of maintaining them. This process was supported by the app features “reminders and nudges” and “checking off daily goals.” Participants felt that they had achieved maintenance when they no longer relied on the app to fulfill their daily goals:

*The meditation, I just go and do that myself. Now I don’t even [use the app] because I’m in the habit of doing it.*

[Participant 13]

The app worked in the background to support participants to stay “on track” while maintaining their habits. Some participants reached a point where they felt confident enough to start introducing new habits due to the strong maintenance process:

*I guess I really should be changing them, the ones that are now coming naturally to me.*

[Participant 11]

Participants acknowledged the challenge of maintenance, whether that be due to forgetfulness, needing further nudges, or the nature of the habit itself. The success of the maintenance process varied, with some habits proving more difficult to maintain than others:

*I try and respond to it daily. I don’t always remember, but I try to. I would say that I’ve been less successful with the exercise things that I’ve set.*

[Participant 11]

###### Increased Sense of Achievement

Often, participants attributed their progress to an increased sense of achievement, facilitated by the features “positive reinforcement,” “tracking and progress reports,” and “setting small goals.” This “sense of accomplishment” served as a powerful motivator, encouraging them to continue progressing with their goals:

*I do like the little confetti you get when you’ve done your habit. You know, I know it’s just psychological, but I quite like the confetti. Yeah, there is a certain satisfaction.*

[Participant 3]

Moreover, highlighting progress milestones provided further gratification, promoting positive reflection and motivation:

*It’s more encouraging. And I also like when it comes up with a kind of big explosion, that’s 100 hours now that you’ve done in the brain studio.*

[Participant 12]

### Psychological Mechanisms: Increased Self-Compassion

Participants emphasized the importance of self-compassion in fostering a more benevolent outlook. This mechanism was linked to the features of “positive reinforcement” and “mindfulness and affirmations.” Many participants described feeling discouraged about incomplete goals and restrictions on what they could achieve due to multimorbidity. This led some participants to “talk poorly” to themselves, using HH to help challenge these negative beliefs and “rationalize.” This was especially effective in prompting participants to acknowledge their progress and establish realistic expectations:

*Since I’ve had Holly Health, I’ve been able to put a lot of things into perspective. Because it’s saying it’s okay to have down days, it’s okay not to feel your best. But it’s also inspiring because it gives you the thought that you can do it another day when you feel better.*

[Participant 6]

By adopting this mindset, HH helped participants cultivate greater self-compassion by not framing experience in absolute terms:

*I think the problem is with my tick charts or where I’ve got a notebook and I’m checking a box or something, I can get down on myself if I’ve bitten off more than I can chew. I’m a bit of a perfectionist I think - avoiding that black and white, you either did it or you didn’t, you were great, or you were rubbish kind of thing.*

[Participant 4]

### Social Mechanisms

#### Increased Feelings of Social Support

Participants described the human-like qualities of HH, which contributed to improving their SWB. This was directly attributed to the “AI interface” feature. Such support was particularly valuable when considering multimorbidity, as interactions with health care professionals are often related to a deterioration in health. The chatbot enabled participants to seek out and experience more preventative and proactive holistic support:

*It almost feels like somebody’s looking out for you, but not in a purely medical context. Because you end most of what you do with the healthcare professional is usually when something goes wrong. So, it’s not when something’s gone wrong, it’s actually slightly helping things not to go wrong.*

[Participant 3]

The impact of the support was especially strong when it was tailored; the “more holistic touch” of HH made the support feel “personal.” This introduced feelings of having friend-like, reliable support:

*It helps with depression because it can talk to you through times of no stimulation and depressive thoughts. It’s like a friend.*

[Participant 6]

#### Reduced Feelings of Loneliness

Living with multimorbidity often led participants to experience feelings of loneliness, with peers sometimes struggling to understand the associated challenges. Feeling listened to and acknowledged was crucial to participants, with some encountering dismissive interactions elsewhere. The “constant support availability” and “AI interface” of HH helped participants to feel less lonely:

*I have chats with them maybe once a week. It does help knowing that somebody is at the other side, even though it’s just a computer, and that somebody’s listening.*

[Participant 16]

Participants expressed that enduring support options were crucial for their well-being, particularly when they found it challenging to actively seek support:

*I do feel Holly (Health) is what’s carried me through that really tough spell. Where I genuinely didn’t really have any support from anyone, and it is much because I couldn’t ask for it.*

[Participant 10]

### Factors Influencing Acceptability of the App

Three factors that affected the acceptability of the app were identified: “type of chronic condition,” “availability of time,” and “using other support tools alongside HH.”

#### Type of Chronic Condition

Many participants viewed HH as a “holistic” support option, capable of addressing a wide range of chronic conditions simultaneously:

*It’s quite universal though, I think there’s so much in there that there’ll be something that would work for anybody’s condition.*

[Participant 11]

However, others noted HH seemed more beneficial for certain conditions, with more advice and support available for general chronic conditions:

*It seems to be designed for quite common conditions, anxiety, depression. I think there are more specific problems I had around rumination […] I don’t think it was specifically good for that, I think it’s good for general wellbeing.*

[Participant 5]

Furthermore, participants with chronic fatigue syndrome (CFS) noted that the app provided limited information and advice on managing their condition, feeling it “needs something extra.” Some felt their conditions were too complex or unique to be fully addressed by HH:

*I don’t really feel that I fit into an easy category. Particularly people with fibromyalgia. Your experience is very individual.*

[Participant 21]

#### Availability of Time

Having time to fully engage in the app and complete the goals was a key factor in how suitable the app was for improving SWB. Many participants valued having the designated time to focus on their well-being goals daily:

*There’s notifications to give you that reminder, because let’s be honest, well life gets in the way of everything, but sometimes you have to take that five-minutes for yourself.*

[Participant 17]

Another participant described that “it’s time for me,” with the app allowing participants to adapt goals to fit within schedules. This was especially apparent in participants who were retired or semiretired, having more time to engage with the app and explore the activities:

*But that’s one of the things about being semiretired and having more time is trying these different things.*

[Participant 9]

#### The Use of Other Support Tools

Many participants used a combination of support to ensure comprehensive care for their mental and physical health, with HH being one component of a broader support regimen:

*Another tool in your general armoury of things. You’re trying to look after yourself as best you can.*

[Participant 15]

Some participants mentioned using multiple apps together, selecting the most suitable based on their current needs:

*I have quite a few different apps on my phone. I suppose I’m always seeking which one feels right in the moment but also will give me longer-term support.*

[Participant 8]

Others combined HH with more intensive forms of support, such as health coaching, adapting techniques from both face-to-face and digital formats to enhance their support plan and maximize the benefits:

*I’m having counselling and stuff anyway, so obviously some techniques from there which I already knew anyway, but it opened my eyes to wider things.*

[Participant 20]

HH was especially valuable when other support tools were not readily available, serving as an interim solution, particularly when waiting for support:

*I was on the wait list again for CBT, so I thought some of the same techniques might be accessible in the app while I’m waiting.*

[Participant 5]

However, some participants found using multiple support tools simultaneously challenging. This limited the extent of the progress made, with some participants perceiving the required commitment as excessive, especially when using multiple health apps together:

*It just felt (it was) difficult to keep doing it because it’s a big commitment to do any one of those apps for a long period.*

[Participant 5]

## Discussion

### Principal Results

This study aimed to examine the impact of a health coaching app on the SWB of individuals with multimorbidity and understand how and why the app supported SWB. Our quantitative analyses revealed that using the app for 8 weeks or longer was associated with increased SWB, including life satisfaction, worthwhileness, happiness, and a reduction in anxiety. There was little evidence that these changes differed by individual characteristics.

Through qualitative analyses, 8 app features were identified as acceptable, categorized by features promoting action, reflective practice, and interaction. These features facilitated a positive impact on SWB through activating behavioral, psychological, and social mechanisms of action. Wider contexts that impact the app acceptability were also identified, including chronic condition type, having time to engage, and the availability of other support tools alongside HH. These findings suggest that a health coaching app can serve as an acceptable support tool within these contexts.

### Comparison With Prior Work

These findings provide evidence for the SWB benefits of a health coaching app for individuals with multimorbidity after 8 weeks or longer. This aligns with previous research demonstrating the effectiveness of digital solutions in enhancing well-being and self-management of single chronic conditions [42,50] alongside previous internal reports from HH, indicating that app usage boosts confidence in managing multimorbidity [51]. Additionally, this is consistent with prior evidence indicating that behavioral interventions can produce measurable well-being improvements within as little as 4 weeks in a range of populations, including people with chronic conditions [52]. Although the estimated changes in SWB scores were modest (<1 point on the Likert scales), such differences are often regarded as meaningful at the population level. For example, the UK HM Treasury Green Book Wellbeing Guidance [53] equates a 1-point increase in life satisfaction with a monetary value of approximately £13,000 (US $17,556.65) per person per year. Furthermore, the observed effect sizes in this study align with those reported in comparable digital behavior change and well-being interventions [54]. While research on digital support tools for multimorbidity is sparse, these findings extend insights from single-condition literature [55], suggesting that this health coaching app may similarly promote self-management and SWB among people with multimorbidity.

The mapping of perceived mechanisms of action to the Multi-Level Leisure Mechanisms Framework [23] enabled the connection between app use and improved SWB to be explored in-depth. These mechanisms often intersect, with behavioral, psychological, and social mechanisms reinforcing one another to support SWB outcomes. Identified behavioral mechanisms of action included maintenance of healthy behaviors and increased sense of achievement. These mechanisms were observed to be supported by reminders and nudges, checking off daily goals, setting small goals, and tracking progress reports. The process involved in checking off daily goals was highlighted as particularly impactful in promoting action, reflected in app users reaching, maintaining, and setting new goals. Positive reinforcement also played a key role in the sense of achievement, encouraging app users to accomplish new goals. This finding is consistent with previous research that suggests goal setting, action planning, and reinforcement are crucial for sustained user engagement and promoting behavior change [56]. Although evidence of the effectiveness of reminders and nudges has previously been inconsistent, the context-specific design of reminders (eg, those tailored to specific habits) was found to be effective in this study. This finding aligns with previous research showing that context-specific reminders are crucial in enabling habit formation [57,58].

Positive reinforcement was also reported to be linked to the psychological mechanism of improved self-compassion. Goal setting similarly facilitated improved self-compassion; app users were able to establish realistic expectations and recognize their progress. This mechanism of action was additionally facilitated by the mindfulness and affirmations content within the DHI, fostering reflective practice. Social mechanisms of action were identified as increased feelings of social support, observed to be activated by the AI interface feature, and reduced feelings of loneliness, reported to be activated by the constant support availability within the DHI. The AI interface was highly valued by app users, and the live chat supported app users through a dependable, personalized approach. These findings are consistent with previous research, with several features identified as valuable in DHIs, including relaxation, personalization, and live support [59]. Distinctly, previous research has recognized increased social support as a key mechanism of improved self-management in multimorbidity interventions [60]. This emphasizes the value of providing features that facilitate this mechanism of action, especially in multimorbidity interventions.

The effect of the DHI can also be strengthened by using evidence-based approaches, such as CBT [35] and the COM-B model [37]. Incorporating these approaches has been found to improve the effectiveness, engagement, perceived quality, and credibility of apps [61-63]. Findings in this study suggest that using app-based support alongside other support tools (eg, health coaching) enhances effectiveness and acceptability. This aligns with previous literature [64], emphasizing the importance of complementary principles to ensure consistency of support.

Novel findings from the qualitative interviews suggest that the complexity of multimorbidity plays a crucial role in the impact and acceptability of the health coaching app as a support tool. While the health coaching app demonstrated a positive impact on SWB, indicating its potential to enhance SWB in people with multimorbidity to some extent, qualitative findings identified that chronic condition type influenced the acceptability of the health coaching app, with lack of information and education on specific conditions consistently a key barrier to support [65]. CFS and fatigue-based symptoms were particularly less accommodated by the app content, which primarily targeted more general conditions such as anxiety. Previous research has emphasized the significance of fear-avoidance beliefs and determining personal thresholds in the efficacy of supporting CFS [66,67]. Therefore, acknowledging these factors is crucial to the acceptability of app-based support for individuals with a CFS comorbidity. Conversely, conditions such as depression and anxiety, which have the highest health care service use in individuals with multimorbidity [24], appear to benefit from the health coaching app as an effective support tool for improving SWB.

The value of DHIs in older adults has been recognized in previous literature, particularly given the high prevalence of long-term conditions in this demographic group [68]. However, there have been concerns that the digital exclusivity of these interventions may lead to higher attrition rates, along with decreased engagement and effectiveness [68]. Notably, our quantitative findings did not identify age as a barrier to SWB improvement, indicating the potential effectiveness of the health coaching app across age groups, including older adults. In fact, our qualitative findings identified that retirement appeared to facilitate greater app engagement, providing individuals with more time to achieve daily goals. This observation aligns with previous research that associates retirement with increased leisure activities, including personal growth and physical exercise [69].

### Strengths and Limitations

The main strength of this study lies in its explanatory sequential mixed methods design, which allowed for a comprehensive understanding of the insights gained from the quantitative phase through qualitative work. This allowed an in-depth exploration of a complex, under-researched area. However, several limitations must be acknowledged. First, the sample in both phases predominantly consisted of White females aged 55 years and older. While this partly reflects the prevalence of multimorbidity in older adults and females [4,5], the lack of diversity in the sample raises concerns about the generalizability of the results to the wider population [70]. Second, confidentiality measures prevented verifying if the same participants were involved in both data collection phases, potentially introducing participant variability and affecting the consistency of results. Third, as participation in the qualitative phase was based on self-selection, the sample may have predominantly consisted of motivated or engaged app users. This self-selection could introduce volunteer bias and limit the generalizability of the findings. However, participants did report varying levels of engagement with the app and spoke about various challenges of use. Similarly, in the quantitative phase, completion of the follow-up questionnaire was optional, which may have resulted in overrepresentation of engaged app users. Additionally, the absence of a control group makes it challenging to establish a causal effect as there is no comparison group against the observed effects, ultimately reducing internal validity. Due to these limitations, caution is advised when interpreting the study findings.

### Conclusions

Our study provides empirical evidence that a health coaching app can be an effective and acceptable support tool to improve the SWB of individuals with multimorbidity. These effects were driven by specific app features promoting action, reflective practice, and interaction. These features led to improved SWB through the activation of reported behavioral, psychological, and social mechanisms. However, the magnitude of these effects could be affected by contextual factors, including users’ time availability for engagement, specific chronic condition profiles, and concurrent use of other support tools. By elucidating the mechanisms and contextual nuances underlying app efficacy, this study provides critical insights to inform the refinement of existing interventions and the design of future DHIs tailored to the complex needs of individuals with multimorbidity.

## Supplementary material

10.2196/78738 Multimedia Appendix 1 Images of the Holly Health app.

10.2196/78738 Multimedia Appendix 2 ONS4 questions and thresholds. ONS4: Office for National Statistics’ 4 personal well-being questions.

10.2196/78738 Multimedia Appendix 3 Interview topic guide.

10.2196/78738 Multimedia Appendix 4 Bayesian unconditional growth curve model technical specifications.

10.2196/78738 Multimedia Appendix 5 Bayesian growth curve model: group effects results.

10.2196/78738 Checklist 1 STROSA and COREQ checklists.

## Abbreviations

AI: artificial intelligence
CBT: cognitive behavioral therapy
CFS: chronic fatigue syndrome
COM-B: Capability-Opportunity-Motivation Behavior Model
COREQ: Consolidated Criteria for Reporting Qualitative Research
DHI: digital health intervention
GP: general practitioner
HDI: highest density interval
HH: Holly Health
NHS: UK National Health Service
NICE: National Institute for Health and Care Excellence
ONS4: Office for National Statistics' 4 personal well-being questions
STROSA: Standardized Reporting of Secondary Data Analyses
SWB: subjective well-being
UCL: University College London
