# Supplementary figures and images for: The Impact of a Health Coaching App on the Subjective Well-Being of Individuals With Multimorbidity: Mixed Methods Study
Source: J Med Internet Res. 2026 Feb 4;28:e78738. doi: 10.2196/78738 (PMC12871578; doi:10.2196/78738)

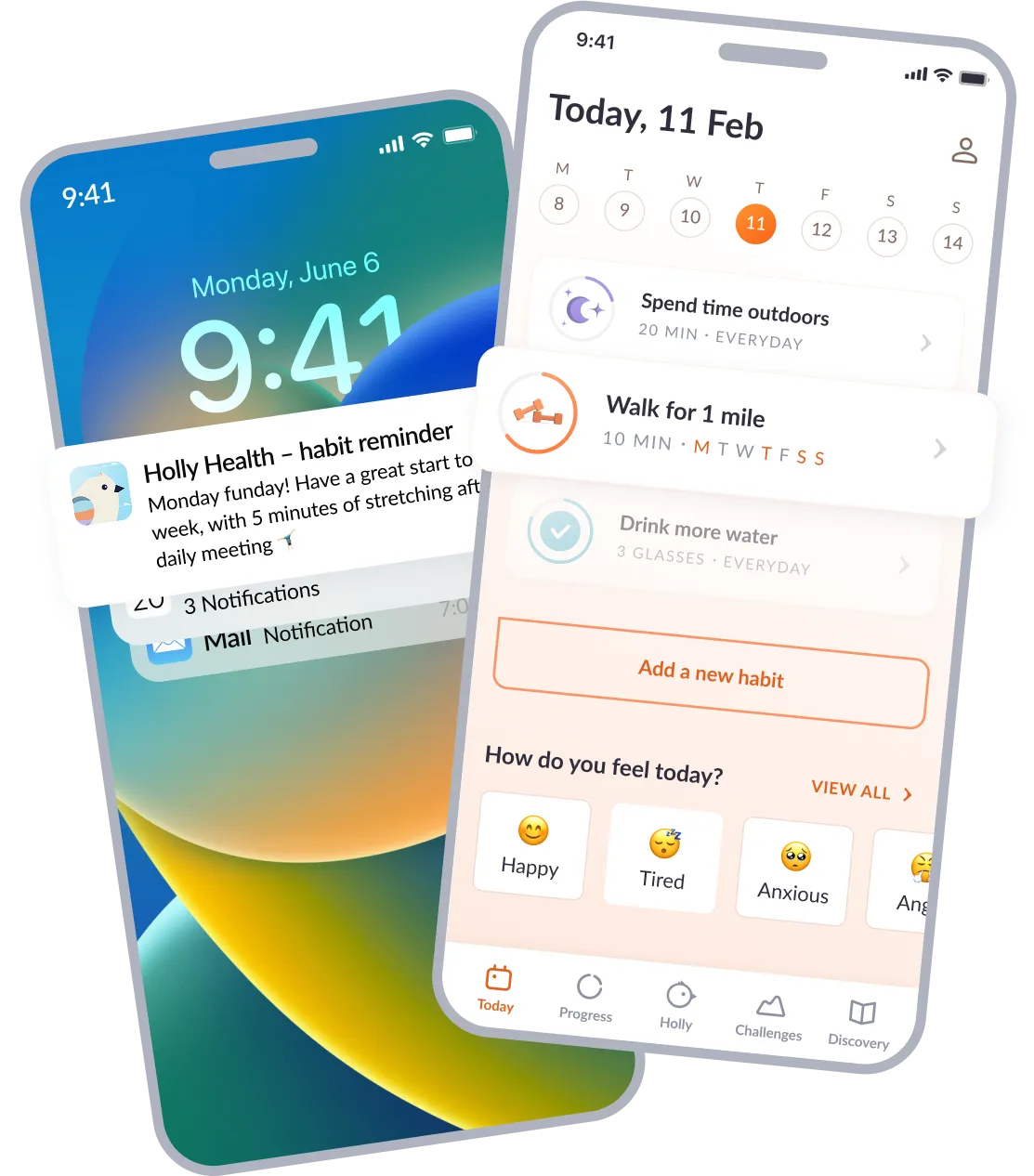


Sourced from: Holly Health | Home. (2023). *Hollyhealth.io.* <https://hollyhealth.io/>

Supplement: Multimedia Appendix 1 [file jmir-v28-e78738-s001.docx]
