# Supplementary material for: The Impact of a Health Coaching App on the Subjective Well-Being of Individuals With Multimorbidity: Mixed Methods Study
Source: J Med Internet Res. 2026 Feb 4;28:e78738. doi: 10.2196/78738 (PMC12871578; doi:10.2196/78738)
Supplement: Multimedia Appendix 2 [file jmir-v28-e78738-s002.docx]

**Table S1.** ONS4 questions.

| Scale of 0-10, where 0 is “not at all” and 10 is “completely”. | |
| --- | --- |
| Measure | **Question** |
| Life Satisfaction | Overall, how satisfied are you with your life nowadays? |
| Worthwhileness | Overall, to what extent do you feel that the things you do in your life are worthwhile? |
| Happiness | Overall, how happy did you feel yesterday? |
| Anxiety | On a scale where 0 is “not at all anxious” and 10 is “completely anxious”, overall, how anxious did you feel yesterday? |

Reference: Rees, E., & Rees, E. (2018). *Personal well-being user guidance - Office for National Statistics*. Ons.gov.uk. <https://www.ons.gov.uk/peoplepopulationandcommunity/wellbeing/methodologies/personalwellbeingsurveyuserguide>

**Table S2.** ONS4 thresholds.

| Life Satisfaction, Worthwhileness, and Happiness Scores | Anxiety Scores |
| --- | --- |
| 11-point scale | **11-point scale** |
| 0-4 = Low | 0-1 = Very low |
| 5-6 = Medium | 2-3 = Low |
| 7-8 = High | 4-5 = Medium |
| 9-10 = Very high | 6-10 = High |

Reference: Rees, E., & Rees, E. (2018). *Personal well-being user guidance - Office for National Statistics*. Ons.gov.uk. <https://www.ons.gov.uk/peoplepopulationandcommunity/wellbeing/methodologies/personalwellbeingsurveyuserguide>
