# Supplementary material for: The Impact of a Health Coaching App on the Subjective Well-Being of Individuals With Multimorbidity: Mixed Methods Study
Source: J Med Internet Res. 2026 Feb 4;28:e78738. doi: 10.2196/78738 (PMC12871578; doi:10.2196/78738)
Supplement: Multimedia Appendix 3 [file jmir-v28-e78738-s003.docx]

Demographics:

1. Age
2. Gender
3. Ethnicity
4. Long-term conditions

Multimorbidity questions:

1. Can you tell me about your experience with having multiple long-term conditions?
   1. *What are some of the challenges you have faced?*
   2. *Do the conditions interact?*
   3. *How do the conditions affect your daily life?*
2. What is your experience with healthcare professionals addressing your long-term conditions?
   1. *Positive or negative?*
   2. *Responsive or dismissive?*
   3. *Barriers to support?*
3. Do you implement any strategies to help cope with your long-term conditions (other than HH)?
   1. *Medication, therapies, support groups, physical activity?*

Holly Health questions:

1. How did you first find out about HH?
2. Why did you decide to use HH?
   1. *Was it specifically related to addressing one condition or multiple?*
   2. *Available through GP?*
3. How has your experience been so far with HH?
   1. *What do you like most about the app?*
   2. *What features do you use the most?*
   3. *Are there any challenges to using the app?*
4. Is it able to accommodate your long-term conditions?
   1. *In what ways?*
   2. *Is it more useful for one condition than another? Why?*
5. What features are most helpful for supporting your long-term conditions?
6. Which features are not helpful?
7. Have you noticed any positive effects since using HH on your long-term conditions and/or psychological well-being?
   1. *PROMPS: What is it about the app that had those impacts?*
8. Have you noticed any negative consequences since using HH?
   1. *PROMPS: What is it about the app that had those impacts?*
