# Supplementary material for: The Impact of a Health Coaching App on the Subjective Well-Being of Individuals With Multimorbidity: Mixed Methods Study
Source: J Med Internet Res. 2026 Feb 4;28:e78738. doi: 10.2196/78738 (PMC12871578; doi:10.2196/78738)
Supplement: Multimedia Appendix 4 [file jmir-v28-e78738-s004.docx]

$$Y_{ij}=\pi_{0i}+\pi_{1i}*{Time}_{ij}+ \varepsilon_{ij}$$

$$\pi_{0i}=\gamma_{00}+ \zeta_{0i}$$

$$\pi_{1i}=\gamma_{10}+ \zeta_{1i}$$

$$\left[ \begin{matrix} \zeta_{0i} \\ \zeta_{1i} \end{matrix} \right]\sim Normal \left( \left[ \begin{matrix} 0 \\ 0 \end{matrix} \right], \left[ \begin{matrix} \sigma_{0}^{2} & \sigma_{01} \\ \sigma_{01} & \sigma_{1}^{2} \end{matrix} \right] \right)$$

$$\varepsilon_{ij} \sim Normal\left( 0, \sigma_{\varepsilon}^{2} \right)$$

Prior specification:

$$\sigma_{0}\sim uniform\left( 0, 100 \right)$$

$$\sigma_{1}\sim uniform\left( 0, 100 \right)$$

$$\rho_{01}\sim uniform\left( -1, 1 \right)$$

$$\sigma_{\varepsilon}\sim uniform\left( 0, 100 \right)$$
