# Supplementary material for: The Impact of a Health Coaching App on the Subjective Well-Being of Individuals With Multimorbidity: Mixed Methods Study
Source: J Med Internet Res. 2026 Feb 4;28:e78738. doi: 10.2196/78738 (PMC12871578; doi:10.2196/78738)
Supplement: Multimedia Appendix 5 [file jmir-v28-e78738-s005.docx]

|  | **Life satisfaction** | | **Worthwhile** | | **Happiness** | | **Anxiety** | |
| --- | --- | --- | --- | --- | --- | --- | --- | --- |
|  | **Coef.** | **95% HDI** | **Coef.** | **95% HDI** | **Coef.** | **95% HDI** | **Coef.** | **95% HDI** |
| Intercept | 5.67 | [5.00,6.33] | 6.27 | [5.65,6.92] | 5.65 | [4.97,6.32] | 4.93 | [4.23,5.69] |
| Slope | 0.56 | [0.05,1.08] | 0.26 | [-0.22,0.77] | 0.76 | [0.26,1.25] | -1.17 | [-1.96,-0.49] |
| Female * Intercept | -0.20 | [-0.94,0.53] | -0.41 | [-1.12,0.31] | -0.10 | [-0.86,0.65] | 0.14 | [-0.67,0.96] |
| Female * Slope | 0.06 | [-0.55,0.56] | 0.29 | [-0.26,0.82] | -0.13 | [-0.72,0.42] | 0.74 | [-0.06,1.62] |
| SD (Intercept) | 2.32 | [2.00,2.72] | 2.19 | [1.89,2.54] | 2.37 | [2.05,2.75] | 2.26 | [1.76,3.03] |
| SD (Slope) | 0.81 | [0.03,1.98] | 0.69 | [0.01,1.88] | 0.76 | [0.02,1.96] | 1.12 | [0.03,3.03] |
| Cor (Intercept, Slope) | -0.38 | [-0.99,0.17] | -0.06 | [-0.68,0.99] | -0.30 | [-0.98,0.37] | -0.13 | [-0.72,0.92] |
| SD Level1 residual | 1.12 | [0.11,1.51] | 1.13 | [0.18,1.49] | 1.21 | [0.32,1.57] | 1.78 | [0.05,2.30] |
| Intercept | 6.25 | [5.77,6.73] | 6.60 | [6.13,7.06] | 6.42 | [5.92,6.92] | 4.63 | [4.08,5.19] |
| Slope | 0.48 | [0.11,0.85] | 0.44 | [0.05,0.79] | 0.51 | [0.12,0.89] | -0.86 | [-1.45,-0.30] |
| Age 18-54 * Intercept | -1.73 | [-2.43,-1.02] | -1.53 | [-2.18,-0.83] | -1.85 | [-2.60,-1.13] | 1.45 | [0.63,2.26] |
| Age 55-64 * Intercept | -0.60 | [-1.28,0.12] | -0.51 | [-1.18,0.16] | -0.81 | [-1.56,-0.12] | -0.08 | [-0.87,0.71] |
| Age 18-54 * Slope | 0.21 | [-0.34,0.75] | 0.00 | [-0.54,0.54] | 0.09 | [-0.50,0.67] | 0.30 | [-0.63,1.09] |
| Age 55-64 *Slope | 0.17 | [-0.38,0.76] | 0.10 | [-0.43,0.63] | 0.32 | [-0.23,0.87] | 0.54 | [-0.28,1.35] |
| SD (Intercept) | 2.16 | [1.85,2.53] | 2.09 | [1.80,2.44] | 2.23 | [1.90,2.64] | 2.10 | [1.63,2.87] |
| SD (Slope) | 0.56 | [0.00,1.83] | 0.69 | [0.01,1.81] | 0.66 | [0.00,2.01] | 0.96 | [0.01,2.90] |
| Cor (Intercept, Slope) | -0.34 | [-1.00,0.46] | -0.06 | [-0.6,0.92] | -0.25 | [-0.99,0.58] | -0.08 | [-0.64,1.00] |
| SD Level1 residual | 1.25 | [0.51,1.58] | 1.17 | [0.44,1.51] | 1.22 | [0.00,1.52] | 1.90 | [0.59,2.40] |
| Intercept | 6.13 | [5.60,6.68] | 6.73 | [6.21,7.25] | 6.14 | [5.58,6.71] | 4.57 | [3.92,5.17] |
| Slope | 0.46 | [0.07,0.88] | 0.18 | [-0.26,0.55] | 0.45 | [0.02,0.88] | -0.86 | [-1.52,-0.25] |
| Conditions 3 * Intercept | -0.33 | [-1.15,0.48] | -0.58 | [-1.34,0.21] | -0.63 | [-1.48,0.22] | -0.08 | [-1.00,0.85] |
| Conditions 4-5 * Intercept | -0.15 | [-0.94,0.64] | -0.53 | [-1.30,0.22] | -0.09 | [-0.92,0.74] | 0.32 | [-0.59,1.21] |
| Conditions 6+ * Intercept | -1.92 | [-2.71,-1.16] | -1.95 | [-2.70,-1.20] | -1.55 | [-2.37,-0.75] | 1.65 | [0.78,2.57] |
| Conditions 3 * Slope | 0.44 | [-0.18,1.09] | 0.50 | [-0.10,1.13] | 0.72 | [0.04,1.38] | 0.57 | [-0.33,1.52] |
| Conditions 4-5 * Slope | -0.01 | [-0.63,0.58] | 0.39 | [-0.27,0.97] | 0.31 | [-0.32,0.94] | 0.27 | [-0.63,1.23] |
| Conditions 6+ * Slope | 0.14 | [-0.45,0.73] | 0.33 | [-0.27,0.93] | -0.15 | [-0.80,0.49] | 0.30 | [-0.59,1.28] |
| SD (Intercept) | 2.15 | [1.84,2.50] | 2.05 | [1.76,2.40] | 2.31 | [1.98,2.70] | 2.06 | [1.60,2.74] |
| SD (Slope) | 0.63 | [0.00,1.70] | 0.65 | [0.00,1.81] | 0.82 | [0.04,2.00] | 0.83 | [0.00,2.63] |
| Cor (Intercept, Slope) | -0.37 | [-1.00,0.48] | -0.02 | [-0.60,0.98] | -0.41 | [-1.00,0.09] | -0.11 | [-1.00,0.76] |
| SD Level1 residual | 1.25 | [0.69,1.53] | 1.18 | [0.42,1.49] | 1.16 | [0.17,1.56] | 1.97 | [1.07,2.39] |
| Intercept | 4.92 | [4.33,5.50] | 5.27 | [4.71,5.84] | 4.53 | [3.94,5.13] | 5.47 | [4.79,6.13] |
| Slope | 0.12 | [-0.33,0.55] | 0.12 | [-0.35,0.57] | 0.82 | [0.37,1.28] | -0.46 | [-1.12,0.21] |
| Auto habit * Intercept | 0.79 | [0.10,1.46] | 0.92 | [0.26,1.57] | 1.38 | [0.67,2.07] | -0.56 | [-1.32,0.22] |
| Auto Habit * Slope | 0.65 | [0.13,1.14] | 0.47 | [-0.08,0.99] | -0.24 | [-0.74,0.29] | -0.16 | [-0.97,0.61] |
| SD (Intercept) | 2.31 | [1.97,2.68] | 2.14 | [1.85,2.51] | 2.28 | [1.97,2.67] | 2.14 | [1.65,2.77] |
| SD (Slope) | 0.86 | [0.00,1.88] | 0.65 | [0.00,1.84] | 0.68 | [0.00,1.93] | 0.92 | [0.00,2.55] |
| Cor (Intercept, Slope) | -0.43 | [-0.98,0.19] | -0.12 | [-0.78,0.79] | -0.29 | [-1.00,0.37] | -0.02 | [-0.57,0.92] |
| SD Level1 residual | 1.12 | [0.33,1.50] | 1.16 | [0.32,1.49] | 1.24 | [0.39,1.58] | 1.96 | [1.15,2.40] |
| Intercept | 5.19 | [4.72,5.65] | 5.59 | [5.15,6.04] | 5.07 | [4.60,5.55] | 5.45 | [4.92,5.98] |
| Slope | 0.45 | [0.10,0.79] | 0.40 | [0.04,0.75] | 0.83 | [0.49,1.18] | -0.91 | [-1.42,-0.38] |
| N habit * Intercept | 0.03 | [0.00,0.07] | 0.04 | [0.00,0.07] | 0.05 | [0.01,0.09] | -0.04 | [-0.08,0.00] |
| N habit * Slope | 0.02 | [-0.01,0.04] | 0.01 | [-0.02,0.04] | -0.02 | [-0.05,0.01] | 0.03 | [-0.01,0.07] |
| SD (Intercept) | 2.33 | [1.97,2.71] | 2.18 | [1.87,2.56] | 2.34 | [2.03,2.71] | 2.21 | [1.75,2.98] |
| SD (Slope) | 0.87 | [0.05,2.00] | 0.80 | [0.03,1.93] | 0.77 | [0.00,1.9] | 1.03 | [0.00,2.96] |
| Cor (Intercept, Slope) | -0.43 | [-0.99,0.07] | -0.09 | [-0.54,0.99] | -0.29 | [-0.98,0.34] | -0.04 | [-0.57,0.99] |
| SD Level1 residual | 1.09 | [0.18,1.51] | 1.07 | [0.00,1.42] | 1.22 | [0.50,1.57] | 1.84 | [0.48,2.38] |
