# Supplementary material for: The Impact of a Health Coaching App on the Subjective Well-Being of Individuals With Multimorbidity: Mixed Methods Study
Source: J Med Internet Res. 2026 Feb 4;28:e78738. doi: 10.2196/78738 (PMC12871578; doi:10.2196/78738)
Supplement: Checklist 1 [file jmir-v28-e78738-s006.docx]

**Table S1.** Standardized Reporting of Secondary Data Analyses (STROSA) checklist.

| **Section** | **Item** | **Recommendation** | **Page** |
| --- | --- | --- | --- |
| **Title and abstract** | | |  |
| Title and abstract | 1 | a) Indicate in the title and abstract that the study is based on secondary data and state the source of the data. | 1 |
|  |  | b) Write a structured summary listing the most important characteristics of the study design, including the study population and case definition. State the secondary data-specific strengths and weaknesses of the work. | 1 |
| **Introduction** | | |  |
| Background and rationale | 2 | Explain the scientific background and rationale for the study presented. Justify your choice of access via secondary data. State the target group and the context of use of your study. | 2-3 |
| Objectives | 3 | State all hypotheses and objectives. Because secondary data analysis is often data-driven, make it clear whether the research question is exploratory or hypothesis-testing. | 2-3 |
| **Methods** | | |  |
| Study design | 4 | Highlight that the study is based on secondary data, what the primary purpose was, and whether you chose a cross-sectional, cohort, case-control, or other study design within your secondary data analysis. | 3-4 |
| Frame | 5 | Identify the data source, its origin, original purpose, data owner, reference population, and time period of the data collected. | 3-5 |
| **Legal basis** | 6 | Explain the contractual and data protection basis on which the data was provided and subsequently analyzed. | 3-5 |
| **Data flow** | 7 | Describe the data flow and indicate who provided the data and where the data analysis was carried out. | 3-5 |
| **Study plan** | 8 | Explain whether your analyses followed a predefined study protocol and to what extent your study is exploratory and/or hypothesis-testing in nature. | 3-5 |
| **Analysis unit** | 9 | Define your units of analysis and clarify whether the data relates to a case and/or a person or insured person. | 3-6 |
| Study participants | 10 | Describe whether a complete survey or a random sample was drawn within the secondary data body. Describe inclusion and exclusion criteria for the inclusion of study participants and, if applicable, matching criteria when using comparison groups. | 4-5 |
| **Internal validations** | 11 | Describe and justify the internal (diagnostic) validation measures taken. | 3-6 |
| Variables | 12 | Clearly define all outcome variables, exposures, predictors, possible confounders and effect modifiers. Explain which of the fields contained in the secondary data body were used and which new variables you derived from them as part of the operationalization. Discuss sociodemographic data available in the secondary data. | 3-6 |
| Classification systems | 13 | Describe which classification systems were used to document illnesses, impairments, medical services, etc. Explain whether and, if so, how the information was validated. | 3-6 |
| Bias | 14 | Discuss the likelihood of selection bias and information bias and the steps you took to determine its existence and magnitude. | 3-6 |
| Study size | 15 | Explain how the study size was determined. Include information on clinical relevance (minimal clinically relevant difference) or relevance for the healthcare system. Justify the use of the entire available data set or a sample-based evaluation. | 5 |
| Quantitative methods | 16 | Describe how you handled counting events such as physician contacts, admissions, number of diagnoses, etc. If necessary, describe how categories (groupings) were formed and why. | 3-6 |
| Statistical methods | 17 | Describe all statistical methods, including those used to control for confounding and to describe subgroups and interactions. Explain how missing data were handled, if any. Describe any sensitivity analyses performed. | 6 |
| **Results** | | |  |
| Selection of the study population | 18 | Describe the process of selection from the original population to the study population clearly, for example in the form of a flow chart. | 3-7 |
| Description of participants | 19 | Describe characteristics of study participants (e.g. demographic, clinical and social characteristics) as well as exposures and possible confounders. Consider whether the study is case- or person-related. | 7 |
| Statistical measures | 20 | Report the number of target events or statistical measures (e.g., mean and standard deviation) or number of participants in each exposure category. | 6-10 |
| Main results | 21 | In addition to raw figures, provide standardized and/or adjusted measures including their precision. If necessary, consider providing estimates of relative and/or absolute risks for meaningful time periods. | 7-10 |
| Further evaluations | 22 | If applicable, report on further evaluations performed, e.g. analysis of subgroups and interactions as well as sensitivity analyses. | 9-11 |
| **Discussion** | | |  |
| Main results | 23 | Summarize the most important results in relation to the study objectives. Before classifying your results, emphasize the secondary data nature of your study. | 16-17 |
| Restrictions | 24 | Comment on the validity of the secondary data in terms of its suitability for answering your research question. Also address information that was not available due to the primary purpose of your secondary data source (e.g. confounding, validity of individual characteristics, consequences of the case definition, exposure and outcome). Discuss the direction and extent of any possible bias. | 16-19 |
| **Strengthen** | 25 | Present the strengths of secondary data analysis in relation to your study, taking into account potentials and limitations. Comment on possible alternative study approaches. | 18-19 |
| Interpretation | 26 | Make a cautious overall interpretation of the results, taking into account the objectives and limitations of the study, the results of other studies, and other relevant evidence. | 16-19 |
| Transferability | 27 | Discuss the transferability (external validity) of the study results to other study populations. | 16-19 |
| **Further information** | | |  |
| Financing | 28 | Indicate how this study was funded and explain the role of the funders in the planning, conduct, analysis, and interpretation of the study. | 19 |
| **Role of data owners** | 29 | Explain the role of the data owners in the planning, conduct, analysis and interpretation of the study and whose responsibility it was for publication. | 19 |

This checklist was developed by: Swart, E., & Schmitt, J. (2014). STandardized Reporting Of Secondary data Analyses (STROSA) - Vorschlag für ein Berichtsformat für Sekundärdatenanalysen. *Zeitschrift Für Evidenz, Fortbildung Und Qualität Im Gesundheitswesen*, *108*(8-9), 511–516. https://doi.org/10.1016/j.zefq.2014.08.022

‌

**Table S2.** Consolidated Criteria for Reporting Qualitative Research (COREQ) checklist.

| **Topic** | **Item No.** | **Guide Questions/Description** | **Page** |
| --- | --- | --- | --- |
| ***Domain 1: Research team and reflexivity*** | | | |
| *Personal characteristics* | | | |
| Interviewer/facilitator | 1 | Which author/s conducted the interview or focus group? | 6 |
| Credentials | 2 | What were the researcher’s credentials? E.g. PhD, MD | 6 |
| Occupation | 3 | What was their occupation at the time of the study? | 6 |
| Gender | 4 | Was the researcher male or female? | 6 |
| Experience and training | 5 | What experience or training did the researcher have? | 6 |
| *Relationship with participants* | | | |
| Relationship established | 6 | Was a relationship established prior to study commencement? | 5-6 |
| Participant knowledge of the interviewer | 7 | What did the participants know about the researcher? e.g. personal goals, reasons for doing the research | 5-6 |
| Interviewer characteristics | 8 | What characteristics were reported about the interviewer/facilitator? e.g. Bias, assumptions, reasons and interests in the research topic | 6 |
| ***Domain 2: Study design*** | | | |
| *Theoretical framework* | | | |
| Methodological orientation and theory | 9 | What methodological orientation was stated to underpin the study? e.g. grounded theory, discourse analysis, ethnography, phenomenology, content analysis | 6-7 |
| *Participant selection* | | | |
| Sampling | 10 | How were participants selected? e.g. purposive, convenience, consecutive, snowball | 5 |
| Method of approach | 11 | How were participants approached? e.g. face-to-face, telephone, mail, email | 5 |
| Sample size | 12 | How many participants were in the study? | 5 |
| Non-participation | 13 | How many people refused to participate or dropped out? Reasons? | 5 |
| *Setting* | | | |
| Setting of data collection | 14 | Where was the data collected? e.g. home, clinic, workplace | 5-6 |
| Presence of nonparticipants | 15 | Was anyone else present besides the participants and researchers? | 5-6 |
| Description of sample | 16 | What are the important characteristics of the sample? e.g. demographic data, date | 5-6 |
| *Data collection* | | | |
| Interview guide | 17 | Were questions, prompts, guides provided by the authors? Was it pilot tested? | 6 |
| Repeat interviews | 18 | Were repeat interviews carried out? If yes, how many? | N/A |
| Audio/visual recording | 19 | Did the research use audio or visual recording to collect the data? | 6 |
| Field notes | 20 | Were field notes made during and/or after the interview or focus group? | N/A |
| Duration | 21 | What was the duration of the inter views or focus group? | 6 |
| Data saturation | 22 | Was data saturation discussed? | N/A |
| Transcripts returned | 23 | Were transcripts returned to participants for comment and/or correction? | N/A |
| ***Domain 3: analysis and findings*** | | | |
| *Data analysis* | | | |
| Number of data coders | 24 | How many data coders coded the data? | 6-7 |
| Description of the coding tree | 25 | Did authors provide a description of the coding tree? | 13 |
| Derivation of themes | 26 | Were themes identified in advance or derived from the data? | 6-7 |
| Software | 27 | What software, if applicable, was used to manage the data? | 6-7 |
| Participant checking | 28 | Did participants provide feedback on the findings? | N/A |
| *Reporting* | | | |
| Quotations presented | 29 | Were participant quotations presented to illustrate the themes/findings? Was each quotation identified? e.g. participant number | 14-16 |
| Data and findings consistent | 30 | Was there consistency between the data presented and the findings? | 13-16 |
| Clarity of major themes | 31 | Were major themes clearly presented in the findings? | 13-16 |
| Clarity of minor themes | 32 | Is there a description of diverse cases or discussion of minor themes? | 13-19 |

This checklist was developed by: Tong A, Sainsbury P, Craig J. Consolidated criteria for reporting qualitative research (COREQ): a 32-item checklist for interviews and focus groups. International Journal for Quality in Health Care. 2007. Volume 19, Number 6: pp. 349 – 357
